# MiR-6869-5p Induces M2 Polarization by Regulating PTPRO in Gestational Diabetes Mellitus

**DOI:** 10.1155/2021/6696636

**Published:** 2021-04-30

**Authors:** Pingping Wang, Zhenzhi Ma, Zengyan Wang, Ximei Wang, Guifeng Zhao, Zengfang Wang

**Affiliations:** ^1^Department of Gynecology and Obstetrics, the Affiliated Hospital of Maternal and Child Health, Weifang Medical University, Weifang 261000, China; ^2^Department of Pharmacy, the First Affiliated Hospital of Weifang Medical University, Weifang 261000, China; ^3^Operating Room, Zhucheng People's Hospital, Zhucheng 262200, China; ^4^Department of Antenatal Diagnosis, the First Affiliated Hospital of Maternal and Child Health, Weifang Medical University, Weifang 261000, China

## Abstract

The role of microRNA (miRNA) in gestational diabetes mellitus has been widely investigated during the last decade. However, the altering effect of miR-6869-5p on immunity and placental microenvironment in gestational diabetes mellitus is largely unknown. In our study, the expression of miR-6869-5p was documented to be significantly decreased in placenta-derived mononuclear macrophages, which was also negatively related to PTPRO. Besides, PTPRO was negatively regulated by miR-6869-5p in placenta-derived mononuclear macrophages. In vitro, miR-6869-5p inhibited macrophage proliferation demonstrated by EdU and CCK-8 experiments. The inflammatory response in macrophages was also significantly inhibited by miR-6869-5p, which could regulate PTPRO as a target documented by luciferase reporter assay. Moreover, miR-6869-5p promoted M2 macrophage polarization and thus restrain inflammation. Accordingly, miR-6869-5p is involved in maintaining placental microenvironment balance by preventing from inflammation and inducing M2 macrophages in gestational diabetes mellitus.

## 1. Introduction

Gestational diabetes mellitus is a common complication in pregnant females, who usually have normal glucose metabolism or potential abnormal glucose tolerance [1]. The incidence of gestational diabetes mellitus has been increasing during the past few decades. Most patients with gestational diabetes mellitus can return to normal glucose metabolism postpartumly, but they are at an elevated risk of developing type II diabetes in the future. The pathogenesis of gestational diabetes mellitus is unclear yet. Most importantly, abnormal glucose metabolism during gestation can cause pregnancy failure, dystocia, stillbirth, fetal death, and fetal macrosomia increased owing to complicated factors [2]. Therefore, identifying useful way for gestational diabetes mellitus prevention and treatment is urgent.

Mononuclear macrophages are key cells involved in regulating placental immunity and homeostasis. Macrophages can be induced to differentiate into classically activated M1 cells and alternatively activated M2 cells under specific regulators and microenvironment in placenta [3, 4]. Type 2 macrophages (M2) play a critical role in maintaining placental microenvironment balance [5]. Accumulated studies have suggested that microRNAs (miRNAs) participate in the pathogenesis of gestational diabetes mellitus by regulating *β* cell development, insulin sensitivity, and resistance [6, 7]. It has been well documented that some miRNAs can affect macrophages differentiation and polarization, such as miR-657, miR-145, and miR-221-3p [8–11]. We have previously found miR-657 could induce macrophages to M1 in gestational diabetes mellitus, which is a promising target for disease diagnosis and treatment [8, 12]. MiR-6869-5p has been firstly found as a cancer suppressor [13]. There is no evidence showing that miR-6869-5p is attributed to gestational diabetes mellitus pathogenesis. We have previously found that the expression of miR-6869-5p was significantly decreased in placenta-derived mononuclear macrophages, which was also negatively related to PTPRO. The bioinformatics analysis has suggested that PTPRO is a potential target of miR-6869-5p. However, little is known about the influence of miR-6869-5p in placental macrophages differentiation and function. In this study, we aim to elucidate the altering effect and potential molecular mechanism of miR-6869-5p on immunity and placental microenvironment in gestational diabetes mellitus, which will provide insight into the disease pathogenesis and gaining promising strategies for disease prevention and therapy.

## 2. Material and Methods

### 2.1. Participants and Samples Preparation

Patients with gestational diabetes mellitus (26 cases) and normal pregnancies (23 controls) are enrolled. Inclusion and exclusion criteria are as follows: pregnant women with GDM having full-term cesarean section are enrolled as the case group, while healthy women having full-term pregnancy are regarded as the control group. Those pregnancies with premature or overdue births are all excluded.  Table 1 lists the characteristics of participants. The study is approved by the hospital's Institutional Ethics Committee of Affiliated Hospital of Maternal and Child Health, Weifang Medical University. Participants have signed the informed consent. Macrophages are freshly separated from placental tissues, which are divided into small pieces before separation. Placental tissue suspension is used for isolating cells by density gradient centrifugation. Then, we used CD14 positive microbeads (Biolegend, USA) for macrophage purification.

### 2.2. Cell Line

Human THP-1 cells are cultured in RPMI 1640 with 10-20% fetal bovine serum (Gibco, USA). PMA (100 nM, Sigma, USA) is used to stimulated cells for 48 h to make them differentiate into macrophages. MiR-6869-5p mimics and miR-6869-5p mimic control are used to coculture with macrophages, which are purchased from Genechem Company (Shanghai, China). Lentivirus plasmids with or without overexpression of protein tyrosine phosphatase receptor type O (PTPRO) are used to transfect macrophages.

### 2.3. CCK-8 and EdU

CCK-8 kit (Vazyme Biotech Nanjing, China) is used to assess cell proliferation. In brief, macrophages (5 × 10^5^/ml per well) with or without overexpression of PTPRO are seeded into 96-well plate for 12 h and, then, are treated by miR-6869-5p mimics and miR-6869-5p mimic control for 24 h and 48 h. 10 *μ*l CCK-8 reagent solution is administrated into cells, which are incubated for 2 h. At last, the optical density (OD) at 450 nm is determined and data are calculated from three independent tests. EdU assay is also performed to estimate macrophage proliferation based on the protocol. Details have been shown in a previous study [8].

### 2.4. Real-Time PCR

Trizol reagent (Invitrogen, USA) is used to isolate RNAs from placental tissue macrophages and THP-1 cells according to the protocol. RNA (0.5 *μ*g) is used as the template for cDNA synthesis based on the protocol of PrimeScriptTM RT Kit (Takara, Beijing, China). cDNA is applied for PCR amplification by use of Takara SYBR Premix. TaqMan PCR assay kit (ThermoFisher Scientific, USA) is used to determine the expression of miR-6869-5p in cells. Primers are as follows: Human PTPRO, F, TATTGTGAGCCTCCGTGTGT; R, GCCAAGCCTTTTCAGTGACA. Human IL-1*β*, F, ACGATGCACCTGTACGATCA; R, TCTTTCAACACGCAGGACAG.

Human TNF-*α*, F, CCCTGAAAACAACCCTCAGA; R, AAGAGGCTGAGGAACAAGCA. Human GAPDH, F, ACCACAGTCCATGCCATCAC, R, TCCACCACCCTGTTGCTGTA.

### 2.5. Enzyme-Linked Immune Sorbent Assay (ELISA)

As previously described [12], THP-1 macrophages with or without overexpression of PTPRO are incubated in serum-free medium for 12 h. Subsequently, macrophages are transfected by miR-6869-5p mimics or mimics control for 48 hours. IL-1*β* and TNF-*α* in cell culture supernatant are determined by ELISA according to the instructions of reagent kits (R & D Systems, USA). The optimal density is finally detected.

### 2.6. Luciferase Reporter Assay

pGL3 vectors carrying the luciferase reporter gene are used to clone the 3' untranscriptional region (3'UTR) of PTPRO (wild and mutant types). The luciferase activity is estimated using the system of dual luciferase reporter assay. Details have been presented in our previous study [8].

### 2.7. Flow Cytometry

Macrophages from placenta tissues of gestational diabetes mellitus patients with high or low expression of miR-6869-5p are incubated with FITC-conjuncted CD14 Ab, and PE-HLA-DR-conjuncted Ab, or PE-CD206-conjuncted Ab (Biolegend, San Diego, CA, USA) at room temperature for 30 min. Cells are then centrifugated and harvested for detection by flow cytometry. THP-1 macrophages (5 × 10^5^/ml) with or without overexpression of PTPRO are seeded into 24-well plate overnight. Then, cells by miR-6869-5p mimics or mimics control for another 24 h. After incubating with PE-HLA-DR-conjuncted Ab or PE-CD206-conjuncted Ab (Biolegend, San Diego, CA, USA) at room temperature for 30 min, we harvest cells and apply flow cytometry for determination.

### 2.8. Statistical Analysis

Mean ± SEM is used for data calculation. All results are normally distributed. The GraphPad Software and SPSS Software are used. Differences between two groups are statistically analyzed by use of independent sample Student's *T*-test for parametric data, while differences among more than three groups are estimated by ANOVA. *P* < 0.05 is considered to be significant.

## 3. Results

### 3.1. MiR-6869-5p Was Significantly Decreased in Macrophages from Placenta and Associated with M2 Macrophages Polarization

**Figure 1:**
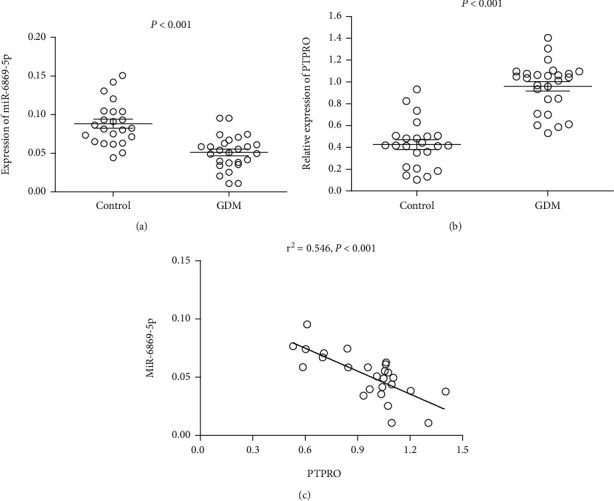
MiR-6869-5p and PTPRO expression in macrophages from placenta. (a) MiR-6869-5p expression in placenta derived macrophages (case/control: 26/23). (b) PTPRO expression in placenta derived macrophages (case/control: 26/23). (c) Association of miR-6869-5p with PTPRO in placenta derived macrophages of gestational diabetes mellitus (GDM) patients (*n* = 26).

**Figure 2:**
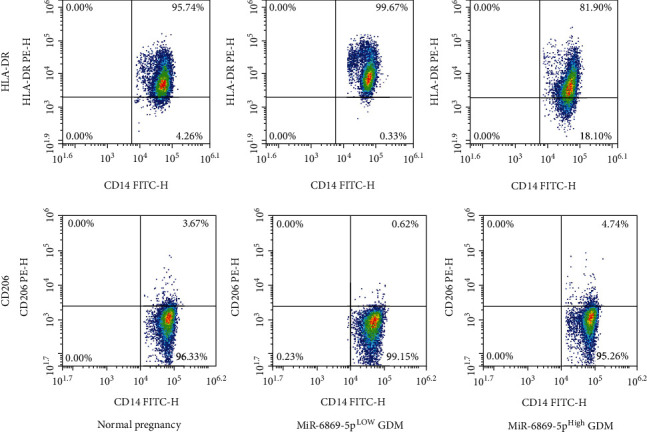
Ratio of CD206^+^ macrophages and HLA-DR^+^ macrophages in placenta of GDM patients compared with normal pregnancies.

The expression of miR-6869-5p in placenta-derived macrophages from gestational diabetes mellitus patients was significantly reduced when comparing with that in placenta-derived macrophages from normal pregnancies (Figure 1(a)). Reversely, increased expression of PTPRO was found in placenta-derived macrophages from patients with gestational diabetes mellitus (Figure 1(b)). Negative association between miR-6869-5p and PTPRO was demonstrated regarding their expression in placental tissue-derived macrophages (Figure 1(c)). We also detected the expression of CD206+ macrophages and HLA-DR^+^ macrophages in placental tissues derived macrophages. Interestingly, CD206^+^ macrophages but not HLA-DR^+^ macrophages were significantly increased in those patients with high expression of miR-6869-5p in placental tissue-derived macrophages, while CD206^+^ macrophages but not HLA-DR^+^ macrophages were significantly decreased in those patients with low expression of miR-6869-5p in placental tissue-derived macrophages (Figure 2). Accordingly, we hypothesize that miR-6869-5p might regulate macrophage polarization in placental immune microenvironment and induces macrophages towards M2 polarization in gestational diabetes mellitus.

### 3.2. MiR-6869-5p Inhibited Macrophage Proliferation by Targeting PTPRO

**Figure 3:**
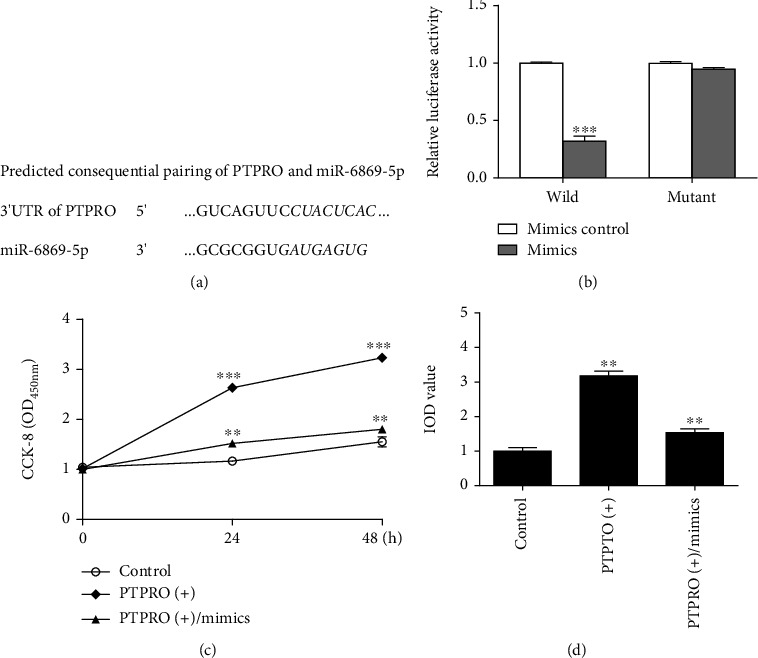
MiR-6869-5p regulated the proliferation of macrophage by targeting PTPRO. (a) The predicted consequential paring of miR-6869-5p and PTPRO 3'UTR. (b) The luciferase reporter assay: the activity of luciferase was significantly decreased in PTPRO wild type group compared with the PTPRO mutant type group in THP-1 macrophages treated by miR-6869-5p mimics (^∗∗∗^*P* < 0.001). (c) CCK-8: miR-6869-5p mimics inhibited the proliferation of macrophage although PTPRO was overexpressed (compared with the control group, ^∗∗∗^*P* < 0.001; compared with the PTPRO (+) group, ^∗∗^*P* < 0.01). (d) EdU: miR-6869-5p mimics inhibited macrophage proliferation (representative pictures of EdU assay and data of three independent experiments; compared with the control group, ^∗∗^*P* < 0.01; compared with the PTPRO (+) group, ^∗∗^*P* < 0.01).

To assure whether miR-6869-5p could regulate PTPRO as a target, we screened the Targetscan database and found that miR-6869-5p could recognize the 3'UTR sequence of PTPRO. The predicted consequential pairing of target region (top) and miRNA (bottom) was shown in Figure 3(a). The luciferase reporter assay further demonstrated our hypothesis that PTPRO was a target gene of miR-6869-5p in THP-1 macrophages (Figure 3(b)). In the following experiments, the altering effects of miR-6869-5p on macrophage proliferation were estimated. As evidenced by CCK-8 (Figure 3(c)) and EdU (Figure 3(d)), the proliferation of THP-1 macrophages could be effectively enhanced when PTPRO was overexpressed in macrophages, while miR-6869-5p mimics could successfully rescue its effect because the proliferation of THP-1 macrophages was significantly inhibited in miR-6869-5p mimics treated group compared with miR-6869-5p mimics control group.

### 3.3. MiR-6869-5p Prevented from Inflammation in Macrophages by Inducing M2 Macrophages

**Figure 4:**
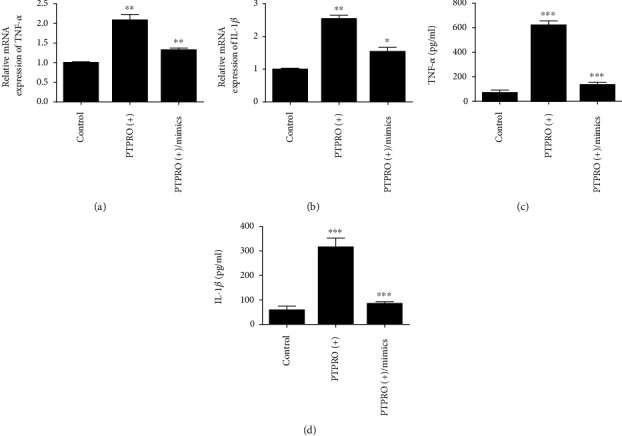
MiR-6869-5p prevented from the production of TNF-*α* and IL-1*β* in macrophages (compared with the control group, ^∗∗^*P* < 0.01, ^∗∗∗^*P* < 0.001; compared with the PTPRO (+) group, ^∗^*P* < 0.05; ^∗∗^*P* < 0.01; ^∗∗∗^*P* < 0.001). (a) mRNA expression of TNF-*α*. (b) mRNA expression of IL-1*β*. (c) TNF-*α* in the supernatant of macrophages. (d) IL-1*β* in the supernatant of macrophages.

**Figure 5:**
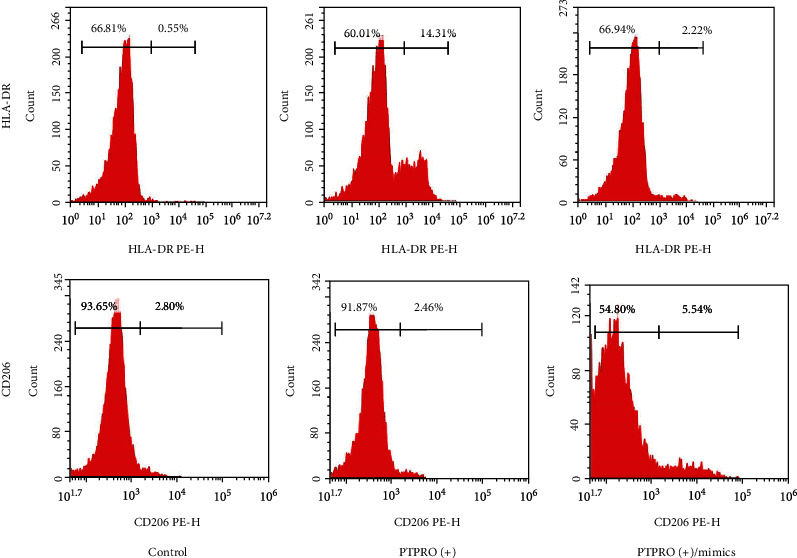
MiR-6869-5p promoted macrophages polarization towards M2.

To estimate the effect of miR-6869-5p on macrophage, THP-1 macrophages were treated by miR-6869-5p mimics and simultaneously stimulated by LPS. As shown in Figures 4(a)–4(d), PTPRO overexpression could induce high expression of TNF-*α* and IL-1*β* at both levels of mRNA and protein in macrophages. Besides, reduced rate of CD206^+^ macrophages and elevated rate of HLA-DR^+^ macrophages was observed when PTPRO was overexpressed in macrophages (Figure 5). However, decreased expression of TNF-*α* and IL-1*β* in macrophages was observed when PTPRO-overexpressed cells were treated by miR-6869-5p mimics (Figures 4(a)–4(d)). Moreover, miR-6869-5p mimic-treated macrophages are more likely to differentiate into CD206^+^ macrophages (Figure 5). Accordingly, miR-6869-5p could rescue the inflammatory response in macrophages induced by PTPRO. Taken together, miR-6869-5p is capable of preventing from inflammation in macrophages by inducing macrophages towards M2.

## 4. Discussion

Gestational diabetes mellitus is a complication of pregnancy, which poses high risks for both the mother and the fetus. The role of noncoding RNAs in gestational diabetes mellitus has drawing more and more attention in the past few years, such as long noncoding RNA and miRNA [14–16]. Although they do not encode active proteins or polypeptides, many noncoding RNAs are involved in the pathogenesis of gestational diabetes mellitus by RNA-RNA or RNA-protein interactions. Some circulating noncoding RNAs can serve as useful disease biomarkers for the onset and progression of gestational diabetes mellitus [17].

miRNA is a small noncoding RNA, which functions by targeting specific mRNAs. A number of miRNAs have been demonstrated to serve crucial roles in gestational diabetes mellitus by protecting pancreatic *β*-cell function, affecting insulin resistance, insulin sensitivity as well as liver gluconeogenesis, for instance, miR-143, miR-351, and miR-96 [18–20]. The study by Yan et al. has reported that miR-6869-5p was dysregulated in colorectal cancer and contributed to cancer cell proliferation, invasion, and migration by negatively regulating TLR4/NF-*κ*B signaling pathway [13]. Exosome-encapsulated miR-6869-5p has also been demonstrated to participate in cancer [21]. However, the modifying effect of miR-6869-5p in macrophages mediated gestational disorders has not been evaluated. In this study, we aim to investigate the miR-6869-5p involvement in gestational diabetes mellitus. MiR-6869-5p is significantly downregulated in placenta derived macrophages from gestational diabetes mellitus patients. It is involved in regulating placental immune microenvironment and inducing macrophages towards M2. MiR-6869-5p prevents from macrophage proliferation and inflammation by targeting PTPRO and promoting macrophages polarization to M2 cells. Accordingly, miR-6869-5p can serve as a suppressor in macrophages mediated inflammatory and immune responses in gestational diabetes mellitus. However, the precise molecular mechanism regarding miR-6869-5p regulation in macrophages proliferation and polarization warrants to be elucidated by more future studies.

Mounting data have implicated that macrophages mediated local immunity plays an important role in maintain the balance of immune microenvironment in placenta [22–24]. Also, macrophages play a critical role in adipose tissue inflammation and immunity [3, 25, 26]. To the best of our knowledge, macrophages can be divided into two common cell types, namely, classically activated M1 and alternatively activated M2 [3, 27]. M1 cells usually possess proinflammation activity, while M2 cells exert anti-inflammation effects. The classic markers for M1 macrophages are CD11c, HLA-DR, and TNF-*α*. The typical markers for M2 macrophages are CD206, CD163, and IL-10. An anti-inflammatory M2 phenotype macrophages is essential for controlling gestational diabetes mellitus [28]. Previously, we have found M1/M2 balance is critical for the maintenance of the maternal-fetal interface immune balance [8]. M1/M2 imbalance would lead to sustained inflammation and immune disorders in the microenvironment of maternal-fetal interface, which may cause premature birth, stillbirth, and so on. Accumulating data have suggested miRNAs participate in the regulating of macrophages proliferation, differentiation, and polarization, which thus contributes to diabetes mellitus [29–31]. Nonetheless, the effect of miRNAs on macrophages polarization in gestational diabetes mellitus is largely unknown. In our study, miR-6869-5p is firstly documented to be positively associated with M2 polarization and protect normal pregnancy in patients with gestational diabetes mellitus. MiR-6869-5p is a promising marker for gestational diabetes mellitus.

PTPRO belongs to protein tyrosine phosphatase family. A number of studies have implicated that PTPRO participates in the regulation of macrophage-mediated inflammatory response, hepatic ischemia reperfusion injury, and tumor immunity [32–35]. In our previous study, PTPRO has been demonstrated to be significantly upregulated in preeclampsia patients, which is also found to be involved in regulating macrophage inflammation in preeclampsia [36]. The current study has firstly suggested that PTPRO is upregulated in placenta-derived macrophages from gestational diabetes mellitus patients. Therefore, we hypothesize that PTPRO may affect the differentiation and function of macrophages and, thus, participate in regulating local immune balance in the placenta. Findings of *in vitro* study have implicated that PTPRO can promote the expression of inflammatory cytokines TNF-*α* and IL-1*β* at both levels of mRNA and protein in macrophages. In addition, PTPRO is capable of inducing macrophages polarization towards M1 and enhancing the inflammatory response. As suggested by the bioinformatics analysis, PTPRO is a targeted gene of miR-6869-5p. MiR-6869-5p might negatively regulate PTPRO at the posttranscriptional level in macrophages. Interestingly, miR-6869-5p can prevent from inflammation by inducing higher expression of CD206 and Arg-1 but lower HLA-DR and CD11c in macrophages. As demonstrated by luciferase reporter assay, the well-established target of miR-6869-5p is PTPRO, a key factor in regulating macrophage mediated inflammation and immune disorders. In general, miR-6869-5p possesses anti-inflammation activity by targetedly regulating PTPRO and inducing M2 macrophages ultimately. Nevertheless, whether miR-6869-5p could exert the same effect *in vivo* needs to be investigated in future studies.

In summary, the present study has identified a miR-6869-5p signature involvement in gestational diabetes mellitus, which may contribute to maintain the balance of placental immune microenvironment by targeting PTPRO and inducing macrophages polarization towards M2.

## Figures and Tables

**Table 1:** Characteristics of patients and controls.

n	Patients	Controls	*P* value
	26	23	
Age (y)	30.6 ± 4.4	29.2 ± 3.5	0.808
Weight (kg)	70.8 ± 5.5	64.6 ± 4.9	0.409
Infant weight (g)	3708 ± 110.2	3528 ± 124.5	0.283
Gestational weeks	38.1 ± 1.2	39.4 ± 1.2	0.449
Blood pressure			
SBP (mmHg)	119.3 ± 11.1	102.4 ± 10.3	0.274
DBP (mmHg)	71.9 ± 7.2	65.2 ± 8.1	0.538
Glucose metabolism			
Fasting glucose (mmol/L)	5.1 ± 1.0	4.2 ± 0.8	0.043
1 h glucose (mmol/L)	10.8 ± 1.7	6.09 ± 0.72	0.019
2 h glucose (mmol/L)	8.5 ± 1.4	5.3 ± 1.1	0.034
Fasting insulin (mIU/L)	9.6 ± 1.1	7.9 ± 1.3	0.320

## Data Availability

Data can be available from upon requesting for the corresponding author.

## References

[1] Chiefari E., Arcidiacono B., Foti D., Brunetti A. (2017). Gestational diabetes mellitus: an updated overview. *Journal of Endocrinological Investigation*.

[2] Szmuilowicz E. D., Josefson J. L., Metzger B. E. (2019). Gestational diabetes mellitus. *Endocrinology and Metabolism Clinics of North America*.

[3] Funes S. C., Rios M., Escobar-Vera J., Kalergis A. M. (2018). Implications of macrophage polarization in autoimmunity. *Immunology*.

[4] Orecchioni M., Ghosheh Y., Pramod A. B., Ley K. (2019). Macrophage polarization: different gene signatures in M1(LPS+) vs. classically and M2(LPS-) vs. alternatively activated macrophages. *Frontiers in Immunology*.

[5] Zhang Y. H., He M., Wang Y., Liao A. H. (2017). Modulators of the balance between M1 and M2 macrophages during pregnancy. *Frontiers in Immunology*.

[6] Chakraborty C., Doss C. G. P., Bandyopadhyay S., Agoramoorthy G. (2014). Influence of miRNA in insulin signaling pathway and insulin resistance: micro-molecules with a major role in type-2 diabetes. *Wiley Interdisciplinary Reviews: RNA*.

[7] Esguerra J. L. S., Nagao M., Ofori J. K., Wendt A., Eliasson L. (2018). MicroRNAs in islet hormone secretion. *Diabetes, Obesity and Metabolism*.

[8] Wang P., Wang Z., Liu G. (2019). miR-657 promotes macrophage polarization toward M1 by targeting FAM46C in gestational diabetes mellitus. *Mediators of Inflammation*.

[9] Roy S. (2016). miRNA in macrophage development and function. *Antioxidants & Redox Signaling*.

[10] Quero L., Tiaden A. N., Hanser E. (2019). miR-221-3p drives the shift of M2-macrophages to a pro-inflammatory function by suppressing JAK3/STAT3 activation. *Frontiers in Immunology*.

[11] Huang Y., Du K. L., Guo P. Y. (2019). IL-16 regulates macrophage polarization as a target gene of mir-145-3p. *Molecular Immunology*.

[12] Wang P., Wang H., Li C. (2019). Dysregulation of microRNA-657 influences inflammatory response via targeting interleukin-37 in gestational diabetes mellitus. *Journal of Cellular Physiology*.

[13] Yan S., Liu G., Jin C. (2018). MicroRNA-6869-5p acts as a tumor suppressor via targeting TLR4/NF-*κ*B signaling pathway in colorectal cancer. *Journal of Cellular Physiology*.

[14] Abdulle L. E., Hao J. L., Pant O. P. (2019). MALAT1 as a diagnostic and therapeutic target in diabetes-related complications: a promising long-noncoding RNA. *International Journal of Medical Sciences*.

[15] Zhang H. (2019). Mechanism associated with aberrant lncRNA MEG3 expression in gestational diabetes mellitus. *Experimental and Therapeutic Medicine*.

[16] Iljas J. D., Guanzon D., Elfeky O., Rice G. E., Salomon C. (2017). Review: bio-compartmentalization of microRNAs in exosomes during gestational diabetes mellitus. *Placenta*.

[17] Guarino E., Delli Poggi C., Grieco G. E. (2018). Circulating MicroRNAs as biomarkers of gestational diabetes mellitus: updates and perspectives. *International Journal of Endocrinology*.

[18] Muralimanoharan S., Maloyan A., Myatt L. (2016). Mitochondrial function and glucose metabolism in the placenta with gestational diabetes mellitus: role of miR-143. *Clinical Science (London, England)*.

[19] Chen S. H., Liu X. N., Peng Y. (2019). MicroRNA-351 eases insulin resistance and liver gluconeogenesis via the PI3K/AKT pathway by inhibiting FLOT2 in mice of gestational diabetes mellitus. *Journal of Cellular and Molecular Medicine*.

[20] Li L., Wang S., Li H. (2018). microRNA-96 protects pancreatic *β*‐cell function by targeting PAK1 in gestational diabetes mellitus. *BioFactors*.

[21] Yan S., Han B., Gao S. (2017). Exosome-encapsulated microRNAs as circulating biomarkers for colorectal cancer. *Oncotarget*.

[22] Zhu X., Liu H., Zhang Z. (2020). MiR-103 protects from recurrent spontaneous abortion via inhibiting STAT1 mediated M1 macrophage polarization. *International Journal of Biological Sciences*.

[23] Jena M. K., Nayak N., Chen K., Nayak N. R. (2019). Role of macrophages in pregnancy and related complications. *Archivum Immunologiae et Therapiae Experimentalis (Warsz)*.

[24] Young O. M., Tang Z., Niven-Fairchild T. (2015). Toll-like receptor-mediated responses by placental Hofbauer cells (HBCs): a potential pro-inflammatory role for fetal M2 macrophages. *American Journal of Reproductive Immunology*.

[25] Jaiswal A., Reddy S. S., Maurya M., Maurya P., Barthwal M. K. (2019). MicroRNA-99a mimics inhibit M1 macrophage phenotype and adipose tissue inflammation by targeting TNF*α*. *Cellular & Molecular Immunology*.

[26] Sisino G., Bouckenooghe T., Aurientis S., Fontaine P., Storme L., Vambergue A. (2013). Diabetes during pregnancy influences Hofbauer cells, a subtype of placental macrophages, to acquire a pro-inflammatory phenotype. *Biochimica et Biophysica Acta*.

[27] Murray P. J., Allen J. E., Biswas S. K. (2014). Macrophage activation and polarization: nomenclature and experimental guidelines. *Immunity*.

[28] Schliefsteiner C., Peinhaupt M., Kopp S. (2017). Human placental Hofbauer cells maintain an anti-inflammatory M2 phenotype despite the presence of gestational diabetes mellitus. *Frontiers in Immunology*.

[29] Zhao Q., Wang X., Hu Q., Zhang R., Yin Y. (2019). Suppression of TLR4 by miR-448 is involved in diabetic development via regulating macrophage polarization. *The Journal of Pharmacy and Pharmacology*.

[30] Zhang M., Zhou Z., Wang J., Li S. (2016). MiR-130b promotes obesity associated adipose tissue inflammation and insulin resistance in diabetes mice through alleviating M2 macrophage polarization via repression of PPAR-*γ*. *Immunology Letters*.

[31] Bouchareychas L., Duong P., Covarrubias S. (2020). Macrophage exosomes resolve atherosclerosis by regulating hematopoiesis and inflammation via MicroRNA cargo. *Cell Reports*.

[32] Zhao J., Yan S., Zhu X., Bai W., Li J., Liang C. (2020). PTPRO exaggerates inflammation in ulcerative colitis through TLR4/NF‐*κ*B pathway. *Journal of Cellular Biochemistry*.

[33] Hou J., Xia Y., Jiang R. (2014). PTPRO plays a dual role in hepatic ischemia reperfusion injury through feedback activation of NF-*κ*B. *Journal of Hepatology*.

[34] Jin K., Liu Y., Shi Y. (2020). PTPROt aggravates inflammation by enhancing NF-*κ*B activation in liver macrophages during nonalcoholic steatohepatitis. *Theranostics*.

[35] Hou J., Deng L., Zhuo H. (2015). PTPROt maintains T cell immunity in the microenvironment of hepatocellular carcinoma. *Journal of Molecular Cell Biology*.

[36] Wang Z., Wang P., Wang Z. (2019). MiRNA-548c-5p downregulates inflammatory response in preeclampsia via targeting PTPRO. *Journal of Cellular Physiology*.

